# Myoinositol Reduces Inflammation and Oxidative Stress in Human Endothelial Cells Exposed In Vivo to Chronic Hyperglycemia

**DOI:** 10.3390/nu13072210

**Published:** 2021-06-27

**Authors:** Maria Pompea Antonia Baldassarre, Pamela Di Tomo, Giorgia Centorame, Assunta Pandolfi, Natalia Di Pietro, Agostino Consoli, Gloria Formoso

**Affiliations:** 1 Department of Medicine and Aging Sciences, G. d’Annunzio University Chieti-Pescara, 66100 Chieti, Italy; marbaldassarre@gmail.com (M.P.A.B.); centogiorgia91@gmail.com (G.C.); consoli@unich.it (A.C.); 2 Center for Advanced Studies and Technology-CAST (ex CeSI Met), G. d’Annunzio University Chieti-Pescara, 66100 Chieti, Italy; pamela.ditomo@unich.it (P.D.T.); assunta.pandolfi@unich.it (A.P.); natalia.dipietro@unich.it (N.D.P.); 3 Department of Medical, Oral and Biotechnological Sciences, G. d’Annunzio University Chieti-Pescara, 66100 Chieti, Italy

**Keywords:** gestational diabetes, HUVEC, endothelial dysfunction, inositol, myoinositol

## Abstract

Myo-inositol (Myo) improves insulin resistance, glucose metabolism, and helps gestational diabetes (GDM) management. GDM is associated with a pro-inflammatory state and increased oxidative stress, which are both involved in vascular damage in diabetes. Our aim was to study Myo anti-inflammatory/antioxidant potential effects on an in vitro model of human umbilical vein endothelial cells (HUVECs). To this end, monocyte cell adhesion to HUVECs, adhesion molecule membrane exposure, and oxidative stress levels were determined in cells from control (C-) and GDM women treated during pregnancy either with diet only (GD-) or with diet plus Myo (GD+Myo). To deeply study the vascular effects of Myo, the same evaluations were performed in C- and GD-HUVECs following 48 h in vitro stimulation with Myo. Notably, we first observed that GD-HUVECs obtained from women assuming Myo supplementation exhibited a significantly decreased number of monocytes that adhered to endothelial cells, less adhesion molecule exposure, and lower intracellular reactive oxygen species (ROS) levels in the basal state as compared to GD-HUVECs obtained from women treated by diet only. This Myo anti-inflammatory/antioxidant effect was confirmed by 48 h in vitro stimulation of GD-HUVECs as compared to controls. Altogether, these results strongly suggest that Myo may exert protective actions against chronic inflammation induced by endothelial dysfunction in diabetes.

## 1. Introduction

Gestational diabetes (GDM) is characterized by insulin resistance, hyperinsulinemia, inflammation, and increased oxidative stress [1].

In GDM, the inflammatory state is characterized by increased plasma circulating levels of pro-inflammatory cytokines, namely tumor necrosis factor-α and interleukin 6 (TNF-α and IL-6, respectively) and lower plasma levels of anti-inflammatory molecules, such as adiponectin and interleukin 10 (IL-10) [2,3]. These patterns are often combined with increased reactive oxygen species (ROS) generation, leading to metabolic alterations and vascular disease [4,5].

Umbilical cord vessels represent a suitable model for the study of vascular alterations, as shown by Di Fulvio and colleagues who characterized the features of human umbilical vein endothelial cells (HUVECs) chronically exposed to hyperglycemia and to a pro-inflammatory environment during pregnancy [5].

Durable pro-atherogenic modifications persist in endothelial cells exposed in vivo, even transiently, to hyperglycemia [5]. Thus, GDM could be viewed as a sort of short-lived metabolic syndrome with oxidative stress-related hyperglycemia and inflammation. In fact, GDM is associated with an increased risk of developing type 2 diabetes and cardiovascular diseases, both in the mother and in the offspring [6].

A low glycemic index diet and a proper lifestyle remain the first approach to treating GDM, and insulin is the only further therapeutic option [7]. As reported recently by the American Diabetes Association (ADA), oral and non-insulin anti-hyperglycemic drugs lack long term safety data, with the exception of metformin, which, however, should be discontinued after the first trimester [7]

In the last decade, convincing evidence has emerged suggesting that inositol may significantly improve maternal and/or fetal outcomes in women with GDM [8]. This appears to be particularly true if the supplement is used early, in the first weeks of pregnancy [9,10,11,12,13,14].

Inositol is a cyclohexanehexol obtainable through biosynthetic mechanisms or from dietary sources. An isoform of inositols, myo-inositol (Myo), is highly concentrated in several foods, such as fruits, beans, grains, and nuts. Lecithin contains the more bioavailable form of inositol [15].

Myo at a daily dose of 4000 mg could be considered for GDM prevention in Caucasian women (Level I, Strength B), as stated in a recent consensus by the Italian Diabetes National Society (SID) based on several recent clinical findings [9,10,11,12,13,16,17,18,19]. The same consensus states that Myo (4000 mg) can be used in Caucasian women for GDM treatment, in association with Nutritional Medical Therapy and lifestyle interventions (Level II, Strength B) [20].

These recommendations are supported by several studies showing that inositol is necessary to activate key enzymes involved in glucose metabolism. Many theories identify inositol as a putative second insulin messenger with anti-inflammatory and insulin sensitizing properties in a variety of experimental models [21].

Limited data are available regarding inositol’s direct effect on vasculature. However, emerging data on preclinical models suggest that inositol might activate a signal pathway leading to nitric oxide (NO) production and exert a superoxide scavenging action [22]. It could therefore prove useful in endothelial alteration prevention and/or treatment. Taking into account that inflammation and oxidative stress are among the main mechanisms involved in insulin resistance and endothelial dysfunction development [23,24], actions aimed at modulating the inflammatory/pro-oxidant diabetes related state should improve, and as a result, insulin resistance and endothelial function should improve, too.

Thus, inositol by its putative anti-inflammatory/anti-oxidant effects could indirectly play a role in alleviating insulin resistance and in improving metabolic disorders and endothelial dysfunction.

Based on these considerations, the aim of the present study was to investigate potential Myo anti-inflammatory/anti-oxidant actions by studying HUVECs obtained from GDM women treated with diet plus Myo supplementation during pregnancy as compared to HUVECs obtained from GDM women treated with diet only. We also aimed to exploit a cellular model to investigate potential Myo anti-inflammatory/antioxidant effects by comparing them to those induced by α-lipoic acid (LA), a molecule with known antioxidant properties.

## 2. Materials and Methods

Dulbecco’s Modified Eagle Medium-low glucose (5.5 mmol/L) (DMEM, CAT. D6046), M199 endothelial growth medium (CAT. M4530), Heparin (CAT. H4415), 0.5% trypsin/0.2% EDTA solution (CAT. 59418C), L-glutamine (CAT. G7513), penicillin-streptomycin (CAT. P4333), phosphate buffered saline (PBS, CAT. D8662), hydrogen peroxide (H_2_O_2_, CAT. H1009), and tumor necrosis factor-α human (TNF-α, CAT. T0157) were purchased from Sigma-Aldrich (Saint Louis, MO, USA). Fetal bovine serum (FBS, CAT. 41A0045K) and CellROX Oxidative Stress Reagents (CAT. C10444) were purchased from Life Technologies Italia (Monza, Italy), and tissue-culture disposables from Eppendorf (Shnelldorf, Germany). Anti-vascular cell adhesion molecule-1 (VCAM-1, CAT. sc-13160) and anti-intercellular adhesion molecule-1 (ICAM-1, CAT. sc-107) antibodies were from Santa Cruz Biotechnology (Santa Cruz, CA, USA). PE-labeled anti-VCAM-1 (CAT. 305806) and FITC-labeled anti-ICAM-1 (CAT. 313104) antibodies were from BioLegend (San Diego, CA, USA).

Myo and LA for in vitro experiments were provided by Laborest Italia (Assago, MI, Italy). The Myo was dissolved in water, while the LA was dissolved in dimethyl sulfoxide (DMSO, CAT. D4540, Sigma-Aldrich), and no dosages exceeded 0.1% in the medium.

### 2.1. Study Design and Clinical Characteristic of Cords Donors

**Scheme 1:**
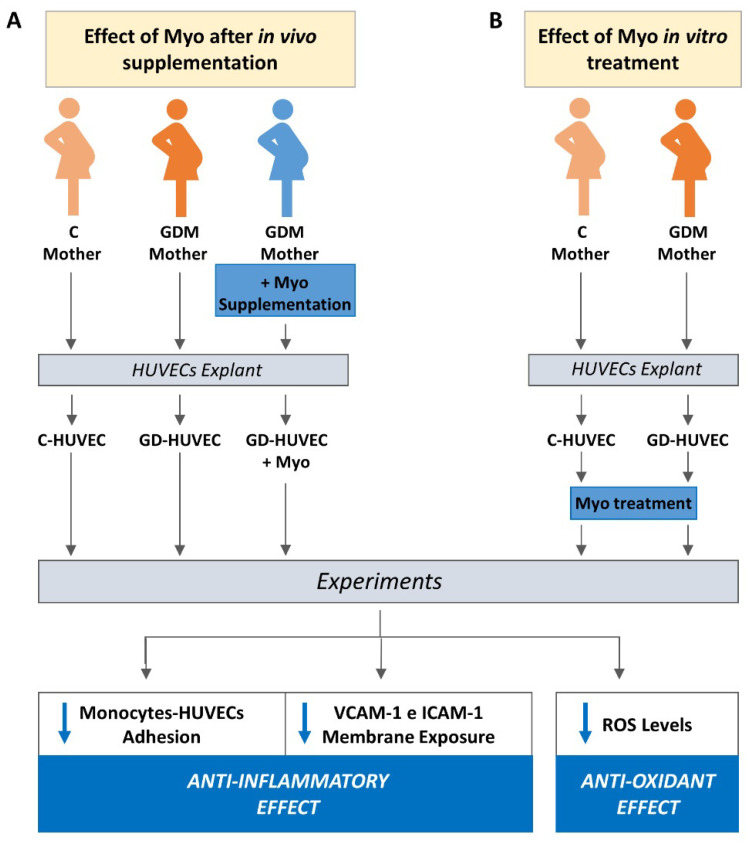
Experimental design of the study and main results of the study. (**A**) We studied HUVEC obtained from non-diabetic (C) and diabetic pregnant women (GDM). Diabetic women were treated with diet alone (GDM) or with diet plus Myo (GDM+Myo). In these three cell groups we conducted the described experiments. This allowed us to assess the characteristics of HUVECs from women supplemented with Myo during pregnancy. The results obtained in this group of cells were compared with the data obtained from HUVECs of non-diabetic women (C) and from diabetic women treated with diet alone (GDM), neither of which received Myo. (**B**) In order to confirm that the characteristics observed in HUVECs exposed in vivo to Myo were specifically due to Myo action, we repeated the indicated experiments in HUVECs harvested from Control and Diabetic women treated with diet only and then exposed them to Myo in vitro only.

The experimental design of the study is shown in Scheme 1.

Umbilical cords were obtained from 10 healthy Caucasian mothers (Control, C) and 16 mothers affected by GDM who delivered at Chieti and Pescara Hospitals (Italy) and who agreed to donate their umbilical cord. Since this is an observational study, it has been conducted following normal clinical practice and all data have been collected retrospectively. Women with pre-gestational diabetes, thyroid dysfunctions, chronic, or systemic diseases as well as GDM women treated with insulin were excluded from analysis. All GDM women were affected by GDM that arose during the second trimester of pregnancy.

Women were treated with a low glycemic index diet and a proper lifestyle. The food plan was based on a nutrition assessment and consisted of three main meals and 3 snacks: the diet was formulated in compliance with the indications provided by Italian Guidelines as follows [25]

- carbohydrates 50% kcal/day (<10% simple sugars);
- lipids 30% kcal/day (<10% saturated fatty acids);
- protein 20% kcal/day (about 0.9 gr/kg/day);
- fibers at least 28 gr/day.

GDM mothers, monitored by the Diabetes and Pregnancy Clinic at the Endocrine and Metabolic Disease Unit, Pescara Town Hospital and University of Chieti-Pescara, were treated with diet only (GD; *n* = 10) or with diet plus supplements containing Myo at a mean dose of 1500 ± 500 mg (GD+Myo; *n* = 6) after the 30th gestational week, as described in Table 1. All procedures were in agreement with the Declaration of Helsinki principles and with the ethical standards of the Institutional Committee on Human Experimentation (Reference Number: 1879/09COET). After approval of the protocol by the Institutional Review Board, signed informed consent was obtained from each participating subject.

### 2.2. Cell Cultures and Experimental Protocols

HUVECs’ explants were obtained and cultured as previously described [26]. For experiments, HUVECs between the third and fifth passage in vitro were grown to sub-confluence in normal growth medium and then were serum-starved (0.5% FBS) and incubated with TNF-α (1 ng/mL), mimicking a low-grade chronic inflammation, for 16 h, following 48 h’ pre-incubation with Myo (0.1–0.5–1 mM) or LA (0.1–0.2 mM) for in vitro treatments. In each experiment we used at least four different cellular strains (*n* = 4) obtained from different umbilical cords of C, GD, or GD+Myo women.

### 2.3. Cell Growth and Viability

C- and GD-HUVECs were seeded at a density of 8000 cells/cm^2^ in twelve-well tissue culture plates and exposed to increasing Myo concentrations (0.025–100 mM) for 48 h. Cell counting was performed by using the Trypan Blue staining method (cat. 15250-061, Life Technologies) in a hemocytometer chamber. We used different cellular strains (*n* = 4) of C- and GD-HUVECs, plating each experimental condition in technical duplicate.

### 2.4. U937 Culture and Adhesion Assays

U937 cell lines (ATCC) were used to evaluate monocyte adhesion to HUVECs’ monolayer. The adhesion assay was performed in the basal state and after stimulation with 1 ng/mL TNF-α for 16 h. For in vitro treatments, C- and GD-HUVECs were previously incubated with Myo (0.1–0.5–1 mM) or LA (0.1–0.2 mM) for 48 h. Cells were grown to confluence in six-well tissue culture plates and U937 cell adhesion was evaluated as previously described [5]. Anti-human monoclonal antibody against VCAM-1 and ICAM-1 was incubated on the cell monolayer 1 h before the assay and used as a control. Adherent cells number was assessed by counting nine different high-power fields (3.5 mm^2^) for each experimental condition in at least three randomly chosen different photos.

### 2.5. VCAM-1 and ICAM-1 Membrane Exposure

To evaluate VCAM-1 and ICAM-1 membrane exposure, non-permeabilized HUVECs were detached by 5 mM EDTA and treated as previously described [27]. Flow cytometry analysis was performed on a BD FACS Canto II flow cytometer (BD Bioscences) and for each sample 1 × 10^4^ events were analyzed using FACSDiva v 6.1.3 (BD Biosciences) and FlowJo 8.3.3 software (Tree Star Inc, Ashland, OR, USA). Results are expressed as MFI (Mean Fluorescence Intensity) ratio, which was calculated by dividing the MFI of positive events by the MFI of negative events (MFI of secondary antibody).

### 2.6. ROS Levels

Five (5) × 10^5^ HUVECs were detached, washed, resuspended in PBS, and incubated with CellROX Oxidative Stress Reagents (cat. C10444, Molecular Probes, Eugene, OR, USA) at 2.5 µM for 30 min at 37 °C. Incubation with 300 µM of H_2_O_2_ for 30 min before the assay was used as a positive control. 1 × 10^4^ events were analyzed by flow cytometry (BD FACS Canto II flow cytometer) as above described.

### 2.7. Statistical Analysis

Data are presented as means ± standard deviation (SD) of at least three independent experiments using different cellular HUVECs explants. Microsoft Excel 2007 and GraphPad software were used for visualization and/or statistical analysis of data. To detect statistically significant differences among HUVEC strains and among different treatments ANOVA tests followed by the recommended test for post hoc comparisons were used. *p* < 0.05 was considered statistically significant.

## 3. Results

### 3.1. Effect of Supplementing GD Mothers with Myo during Pregnancy on GD-HUVECs In Vitro Features

**Figure 1:**
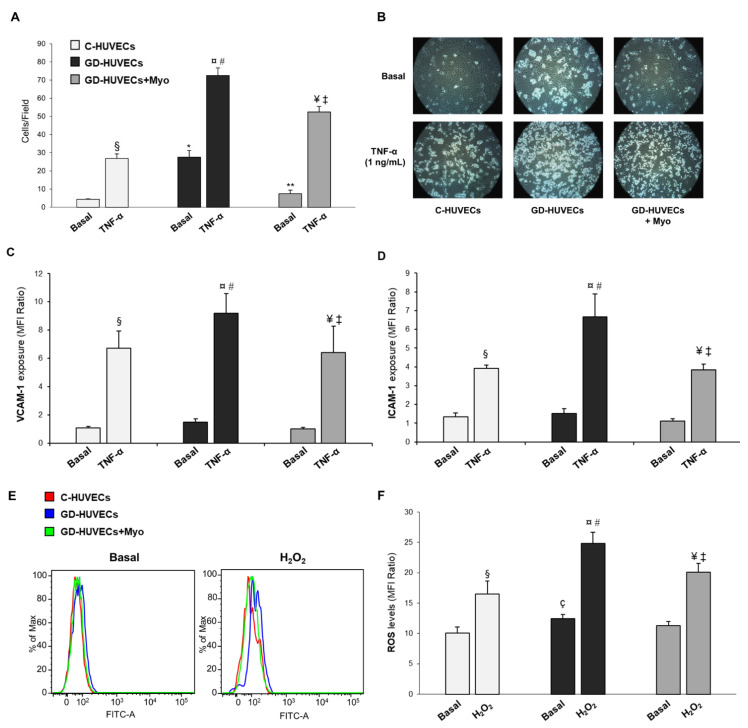
Effect of supplementing GD mothers with Myo during pregnancy on GD-HUVECs in vitro features. (**A**) Effect of in vivo treatment with Myo on U937 monocyte adhesion to the endothelium. (**B**) Representative pictures of C-, GD- and GD+Myo-HUVECs for each experimental condition. (**C**,**D**) Effect of in vivo Myo treatment on adhesion molecule membrane exposure (VCAM-1, panel C and ICAM-1, (**D**). (**E**) Representative histograms obtained from the cytometric analysis for Basal condition and after H_2_O_2_ treatment. (**F**) Effect of Myo in in vivo treatment on ROS levels in endothelial cells. * *p* < 0.01 vs. Basal C-HUVECs; ** *p* < 0.01 vs. Basal GD-HUVECs; § *p* < 0.05 vs. Basal C-HUVECs; ¤ *p* < 0.05 vs. Basal GD-HUVECs; ¥ *p* < 0.05 vs. Basal GD-HUVECs + Myo; # *p* < 0.05 vs. TNF-α C-HUVECs; ‡ *p* < 0.05 vs. TNF-α GD-HUVECs; ç *p* < 0.05 vs. Basal C-HUVECs.

Monocyte (U937) adhesion, adhesion molecule membrane exposure, and ROS levels of HUVECs obtained from diabetic women treated during pregnancy with diet only (GD-HUVECs) or diet plus Myo supplementation (GD-HUVECs+Myo) are reported in Figure 1 (and Table S1 in the Supplementary Materials).

In GD-HUVECs+Myo, the number of U937 cells that adhered to the cell monolayer was significantly lower both in the basal state (*p* < 0.01) and after TNF-α stimulation as compared to GD-HUVECs (*p* < 0.05) (Figure 1A,B).

As shown in Figure 1C,D, VCAM-1 and ICAM-1 TNF-α induced membrane exposure was significantly lower in GD-HUVECs+Myo as compared to GD-HUVECs (*p* < 0.01). Finally, as shown in Figure 1E,F, GD-HUVECs+Myo exhibited lower intracellular ROS levels both in the basal state and after H_2_O_2_ stimulation as compared to GD-HUVECs (*p* < 0.05).

### 3.2. Effect of Myo on TNF-α Stimulated U937 Monocyte Adhesion to C- and GD-HUVECs

We used Myo concentrations of 0.1, 0.5, and 1 mM in subsequent experiments without affecting cell viability (Figure S1). Corresponding doses of DMSO in the medium for all dosages did not exceed 0.1%. MTT experiments were carried out also with the vehicle alone showing no differences in cell viability (data not shown).

**Figure 2:**
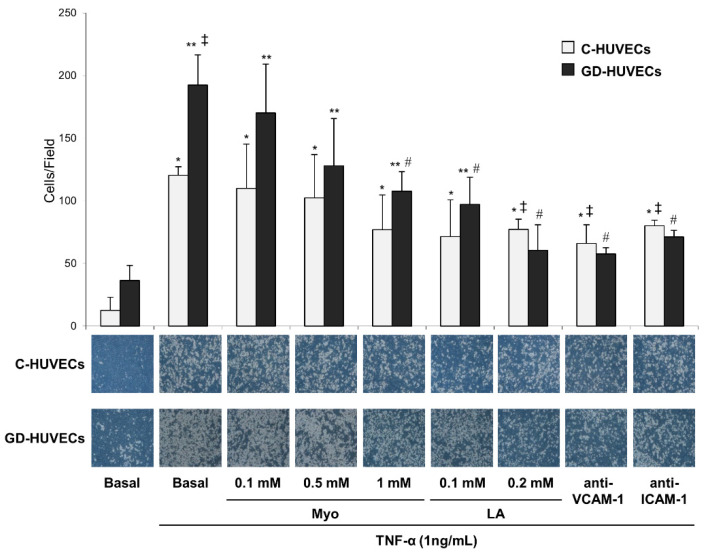
Effect of in vitro treatment with Myo on TNF-α-induced monocyte interaction in C- and GD-HUVECs. In the lower side are representative photos of C- and GD-HUVECs for each experimental condition. * *p* < 0.01 vs. Basal C-HUVECs, ** *p* < 0.005 vs. Basal GD-HUVECs, ‡ *p* < 0.05 vs. TNF-α C-HUVECs, # *p* < 0.05 vs. TNF-α GD-HUVECs.

Monocyte (U937) adhesion to C-HUVECs and GD-HUVECs was studied in the basal state and after TNF-α stimulation (1 ng/mL for 16 h) with 48 h Myo (0.1–0.5–1 mM) pre-incubation (Figure 2 and Table S2 in the Supplementary Materials). As previously observed [5,26,28], we confirmed that TNF-α stimulation dramatically increased the number of U937 cells that adhered to the HUVECs monolayer in C and GD cellular models (*p* < 0.01 and *p* < 0.005, respectively); this effect was, however, significantly more prominent in GD-HUVECs than in C-HUVECs (*p* < 0.05).

It is important to note that pre-treatment with Myo induced a trend toward a reduction in monocyte adhesion in both cell types, which was particularly evident and statistically significant in GD-HUVECs exposed to 1 mM Myo (*p* < 0.05). This was similar to what was observed in HUVEC pre-incubated with 0.2 mM LA, which also significantly reduced U937 adhesion to HUVECs-monolayer in both C- and GD-HUVECs (*p* < 0.05).

Anti-VCAM-1 or anti-ICAM-1 antibody treatment at saturating concentrations led to a significant decrease of U937 adhesion to both C- and GD-HUVECs (*p* < 0.05), indicating that adhesion molecules play a crucial role in increasing U937 adhesion to HUVECs.

### 3.3. Effect of Myo on TNF-α Stimulated Adhesion Molecule Membrane Exposure in C- and GD-HUVECs

**Figure 3:**
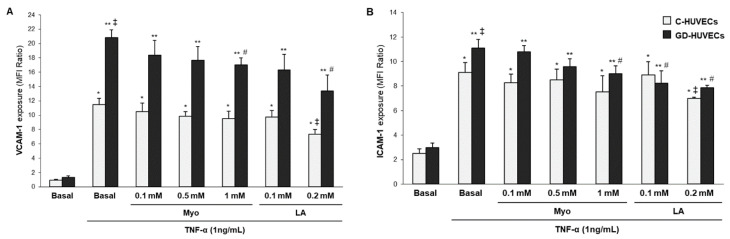
Effect of in vitro treatment with Myo on VCAM-1 (**A**) and ICAM-1 (**B**) membrane exposure after TNF-α-stimulation in C- and GD-HUVECs. * *p* < 0.001 vs. Basal C-HUVECs, ** *p* < 0.001 vs. Basal GD-HUVECs, ‡ *p* < 0.05 vs. TNF-α C-HUVECs, # *p* < 0.05 vs. TNF-α GD-HUVECs.

As shown in Figure 3 (and Table S3 in the Supplementary Materials), after TNF-α stimulation VCAM-1 and ICAM-1 membrane exposure significantly increased (*p* < 0.001) in both C- and GD-HUVECs. Moreover, this effect was more pronounced in GD-HUVECs (*p* < 0.05) as compared to C-HUVECs, as we previously reported [26,27,29]. Pre-treating GD-HUVECs with Myo 1 mM induced a significant reduction in both VCAM-1 and ICAM-1 exposure (*p* < 0.05).

Pre-treatment with LA 0.2 mM significantly reduced VCAM-1 and ICAM-1 TNF-α induced membrane exposure in both cellular models (*p* < 0.05).

### 3.4. Effect of Myo on Intracellular ROS Levels in GD HUVECs

**Figure 4:**
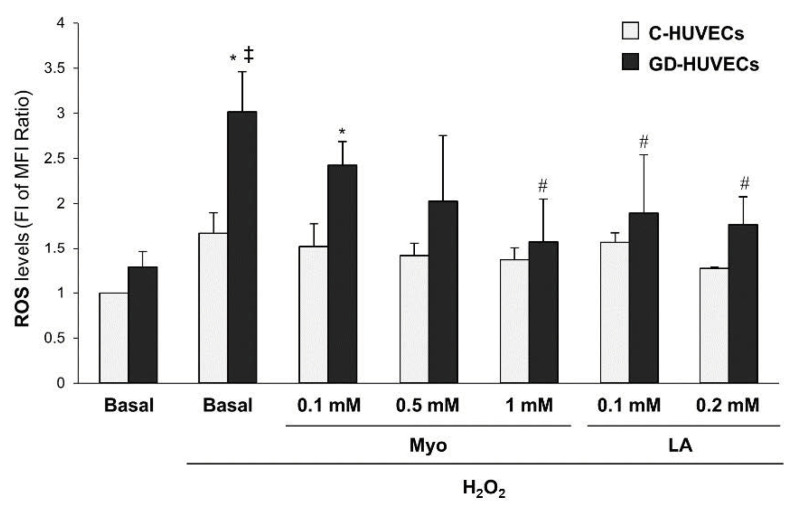
Effect of in vitro treatment with Myo on ROS levels in C- and GD-HUVECs. * *p* < 0.005 vs. Basal GD-HUVECs, ‡ *p* < 0.005 vs. H_2_O_2_ C-HUVECs, # *p* < 0.05 vs. H_2_O_2_ GD-HUVECs.

As shown in Figure 4 (and Table S4 in the Supplementary Materials), at baseline intracellular ROS levels were nominally higher in GD-HUVECs as compared to C-HUVECs. After H_2_O_2_ stimulus, a significant increase in intracellular ROS levels was observed only in GD-HUVECs (*p* < 0.005), which was significantly blunted by pre-treatment with Myo 1 mM or LA (*p* < 0.05).

## 4. Discussion

In the present study, we used a cellular model to investigate Myo actions potentially related to a putative role in the prevention of diabetes-related cardiovascular damage. We employed a human vascular cell model, namely endothelial cells deriving from umbilical cords (i.e., HUVECs), which represent a cellular model of endothelial dysfunction occurring during hyperglycemic conditions. By using this experimental model, we were able to demonstrate a definite anti-inflammatory and antioxidant effect of Myo in human cells, allowing us to hypothesize that it could exert protective actions against chronic inflammation induced by endothelial dysfunction in diabetes.

Specifically, we evaluated the potential Myo direct anti-inflammatory and antioxidant effects in HUVECs obtained from pregnant women with GDM supplemented with Myo compared to cells from GDM women treated with diet therapy alone (Scheme 1). Moreover, in order to enforce the data obtained in vivo, we studied cells exposed in vitro to this molecule (Scheme 1). Thus, cells were exposed in vitro to Myo concentrations comparable to circulating Myo levels following oral supplementation in vivo in humans: these concentrations did not prove toxic for the cells.

As to Myo’s anti-inflammatory and anti-oxidant actions, we found that Myo, supplemented in vivo, significantly reduced levels of monocyte cell adhesion, adhesion molecule exposure, and intracellular ROS levels in the basal state as compared to GD-HUVECs obtained from women treated by diet-only.

It is worth noting that the positive changes induced by Myo supplementation in vivo persist in vitro after cell explant and culture, suggesting that Myo could act as a modulator of ‘endothelial cell memory’ of inflammation induced by GDM.

*In vitro,* Myo significantly reduced monocyte-HUVECs adhesion induced by TNF-α, an inflammatory insult, in C- and GD-HUVECs. We also observed that Myo significantly decreased TNF-α-induced VCAM-1 and ICAM-1 membrane exposure in both control and GD cells. Myo was able to significantly control ROS production in GD cells while only a trend toward this direction was observed in control cells. Since both in the basal state and after pro-oxidant insult, oxidative stress was significantly greater in GD- as compared to C-HUVECs, it is possible (and indeed very likely) that the Myo effect was clearly detectable only in GD-HUVECs because of the presence of an enhanced pro-oxidative state.

Overall, these data represent the first evidence that Myo is able to improve endothelial stress/damage in vascular cells by modulating inflammation and oxidative stress related to diabetes. These effects are comparable to the protective vascular action of LA, a molecule with well-known antioxidant effects that has demonstrated safety as well as beneficial effects on glycemia and liver enzymes as well as on lipid profiles and on adiponectin/leptin ratios in women affected by either GDM or polycystic ovary syndrome [8,27,30,31,32,33].

It is widely accepted that insulin resistance and hyperglycemia, closely associated with chronic inflammation and enhanced oxidative stress, are among the main factors causing vascular damage in women with GDM [34].

Alterations such as increased leukocyte adhesion molecule expression or increased intracellular ROS levels are well documented in endothelial cells harvested from the umbilical cords of women affected by GDM and are thought to be related to chronic low grade inflammation and hyperglycemia [5].

It is important to note that different inositol-containing compounds are derived from Myo metabolism, which are involved in many critical cell activities such as insulin signal transduction, membrane biogenesis, vesicle trafficking, and chromatin remodeling [35]. Accordingly, inositol seems to be able to positively modulate glucose metabolism [8]. Indeed, the advantage of inositol is documented by several clinical findings suggesting that, if used early in the course of pregnancy in women at high risk for GDM, it is able to significantly reduce the incidence of glucose metabolism alterations during pregnancy [9,10,11,12,13,16,17,18,19].

Although Myo is contained in several foods in a balanced diet, it is convenient to provide by supplement in order to reach the proper daily dosage.

Indeed, two grams of Myo twice a day in GDM seems to be associated with improved maternal glucose metabolism and reduced adverse foetal/neonatal outcomes [8].

According to a recent study by Pillai and colleagues, placental inositol content was lower in GDM as compared with non-GDM, with an inverse correlation between maternal blood glucose and placental inositol levels [36]. The biological implications of reduced placental inositol levels in GDM are unclear; however, the authors suggested that a higher placental inositol content may attenuate the foetal growth–adipogenic effects of maternal glycemia as observed in the Singapore GUSTO mother-offspring cohort [37]. Thus, inositol supplementation during pregnancy might increase placental inositol levels and reduce the risk of foetal macrosomia [36].

A number of studies have documented Myo’s anti-inflammatory and insulin-sensitizing properties in a variety of experimental models, pointing to a possible significant role for this molecule in modulating molecular pathways leading to inflammation, oxidative stress, and insulin resistance [8,21]. In a preclinical murine model, Myo plays a protective role against stress-induced macrophage metabolism, resulting in a favourable environment for these cells, finally providing an adequate anti-inflammatory response [38]. Moreover, in a rat model of insulin resistance, a reduction in the pro-inflammatory cytokine IL-6 circulating levels was observed after Myo supplementation [39]. Interestingly, Myo treatment has been shown to increase synthesis and function of surfactant, improving lung immune defense response [40].

Nascimento and collaborators showed that inositol supplementation improves endothelial function in vessels from diabetic rat and rabbit [22]. The same authors also observed that D-Chiro-Inositol (an isomer of Myo) acutely reduced oxidative stress and preserved nitric oxide signalling in endothelial cells [22]. The positive D-Chiro-Inositol effects on vascular damage progression could be mediated by modulation of the protein kinase C (PKC) activation, hexosamine pathway activity, and advanced glycation end product formation [22].

Although, in rat models, Myo treatment seems not to protect microvascular vessels (glomerular and retinal vasculature) from diabetes-induced changes [41], D’Oria and colleagues showed in human endothelial cells that inositols activate PKB/Akt and MAPK/ERK pathways, potentially promoting cell survival and cellular metabolism [42].

In conclusion, implementing strategies aiming to improve the intrauterine environment in women with diabetes is a useful tool that will allow for the control of epigenetic modifications of foetal tissues and the associated cardiovascular risk of new-borns [34,43].

Since in most countries none of the oral anti-hyperglycemic drugs are approved for use during pregnancy, supplementation with Myo could therefore represent a valid option to reduce the development of diabetes and to control the future onset of cardiovascular disease in these women as well as in the next generation.

## 5. Conclusions

Myo is able to improve endothelial stress/damage in vascular cells by modulating inflammation and oxidative stress related to diabetes.

## Figures and Tables

**Table 1:** Characteristics of women from whom umbilical cords were obtained and birth weight of newborns at delivery.

Characteristics	Control (*n* = 10)	GD (*n* = 10)	GD+Myo (*n* = 6)
Age (years)	34.6 ± 4.93	39 ± 8.74 *	41 ± 5.45 *
Pre-gestational weight (kg)	63.05 ± 14.47	67.1 ± 10.73	64.75 ± 13.15
Pre-gestational BMI (Kg/m^2^)	25.12 ± 4.60	26.8 ± 3.10	26.2 ± 3.70
Weight at delivery (kg)	73.05 ± 14.47	79.23 ± 16.32	77.75 ± 13.18 #
Gestational week OGTT	28.1 ± 2.85	25 ± 2.64 *	25 ± 8.08 *
Glycemia T0 (mmol/L)	4.5 ± 0.24	5.1 ± 0.24 *	5.2 ± 1.26 *
Glycemia T60 (mmol/L)	8.1 ± 0.99	10.2 ± 1.16 *	11.3 ± 1.59 *
Glycemia T120 (mmol/L)	6.54 ± 1.14	8.04 ± 1.71 *	9.8 ± 1.51 *
Systolic BP (mmHg)	110 ± 20	100 ± 28.28	110 ± 8.54
Diastolic BP (mmHg)	75 ± 15	75 ± 21.21	70 ± 7.07
Treatment	Diet	Diet	Diet + Myo (1500 ± 500 mg)
Gestational week at delivery	38.7 ± 1.40	38.5 ± 1.52	38.5 ± 1.60
Birth Weight (g)	3408.50 ± 589.50	3490.30 ± 501.50	3475.35 ± 473.10

GD, gestational diabetes treated with diet; GD+Myo, gestational diabetes treated with diet plus supplements containing Myoinositol; BMI, body mass index; OGTT, oral glucose tolerance test; BP, blood pressure. Data are presented as means ± standard deviation; * *p* < 0.05 vs. Control; # *p* < 0.05 vs. GD.

## Supplementary Materials

The following are available online at https://www.mdpi.com/article/10.3390/nu13072210/s1, Table S1: Values and significance of Figure 1; Table S2: Values and significance of Figure 2; Table S3: Values and significance of Figure 3; Table S4: Values and significance of Figure 4; Figure S1: Effect of in vitro treatment with Myo on viability in C- and GD-HUVECs.
