# Supplementary material for: Myoinositol Reduces Inflammation and Oxidative Stress in Human Endothelial Cells Exposed In Vivo to Chronic Hyperglycemia
Source: Nutrients. 2021 Jun 27;13(7):2210. doi: 10.3390/nu13072210 (PMC8308270; doi:10.3390/nu13072210)
Supplement: Supplementary file 1 [file nutrients-13-02210-s001.zip › nutrients-1256763-supplementary.pdf]

Supplementary Materials:

Table S1. Values and significance of Figure 1.

Panel A	C-HUVECs	GD-HUVECs	GD-HUVECs+Myo	p
**Basal**	4.33 ± 0.28	27.5 ± 3.68 *	7.5 ± 1.89 **	* < 0.01 vs C-HUVECs<br>** < 0.01 vs GD-HUVECs
**TNF-α**	26.75 ± 2.63 §	72.5 ± 4.2 ¤ #	52.4 ± 3.08 ¥ ‡	§ < 0.05 vs Basal C-HUVECs<br>¤ < 0.05 vs Basal GD-HUVECs<br>¥ < 0.05 vs Basal GD-HUVECs+Myo<br># < 0.05 vs C-HUVECs<br>‡ < 0.05 vs GD-HUVECs
**Panel C**	**C-HUVECs**	**GD-HUVECs**	**GD-HUVECs+Myo**	**p**
**Basal**	1.06 ± 0.09	1.48 ± 0.26	1.0 ± 0.12	n/a
**TNF-α**	6.7 ± 1.21 §	9.19 ± 1.4 ¤ #	6.4 ± 1.86 ¥ ‡	§ < 0.05 vs Basal C-HUVECs<br>¤ < 0.05 vs Basal GD-HUVECs<br>¥ < 0.05 vs Basal GD-HUVECs+Myo<br># < 0.05 vs C-HUVECs<br>‡ < 0.05 vs GD-HUVECs
**Panel D**	**C-HUVECs**	**GD-HUVECs**	**GD-HUVECs+Myo**	**p**
**Basal**	1.34 ± 0.2	1.53 ± 0.23	1.12 ± 0.11	n/a
**TNF-α**	3.92 ± 0.16 §	6.67 ± 1.22 ¤ #	3.85 ± 0.29 ¥ ‡	§ < 0.05 vs Basal C-HUVECs<br>¤ < 0.05 vs Basal GD-HUVECs<br>¥ < 0.05 vs Basal GD-HUVECs+Myo<br># < 0.05 vs C-HUVECs<br>‡ < 0.05 vs GD-HUVECs
**Panel F**	**C-HUVECs**	**GD-HUVECs**	**GD-HUVECs+Myo**	**p**
**Basal**	10.04 ± 1.04	12.46 ± 0.7 ç	11.26 ± 0.7	ç < 0.05 vs Basal C-HUVECs
**H_2O_2**	16.53 ± 2.09 §	24.8 ± 1.85 ¤ #	20.08 ± 1.47 ¥ ‡	§ < 0.05 vs Basal C-HUVECs<br>¤ < 0.05 vs Basal GD-HUVECs<br>¥ < 0.05 vs Basal GD-HUVECs+Myo<br># < 0.05 vs C-HUVECs<br>‡ < 0.05 vs GD-HUVECs

Table S2. Values and significance of Figure 2

	C-HUVECs	GD-HUVECs	p
**Basal**	12.58 ± 10.5	36.29 ± 12.04	n/a
**TNF-α**	120.37 ± 6.8 *	192.5 ± 23.9 ** ‡	* < 0.01 vs Basal C-HUVECs<br>** < 0.005 vs Basal GD-HUVECs<br>‡ < 0.05 vs TNF-α C-HUVECs
**TNF-α +<br>Myo 0.1 mM**	109.92 ± 35.4 *	170.19 ± 39.1 **	* < 0.01 vs Basal C-HUVECs<br>** < 0.005 vs Basal GD-HUVECs
**TNF-α +<br>Myo 0.5 mM**	102.29 ± 34.5 *	127.9 ± 37.7 **	* < 0.01 vs Basal C-HUVECs<br>** < 0.005 vs Basal GD-HUVECs
**TNF-α +<br>Myo 1 mM**	77.05 ± 27.6 *	107.6 ± 15.7 ** #	* < 0.01 vs Basal C-HUVECs<br>** < 0.005 vs Basal GD-HUVECs<br># < 0.05 vs TNF-α GD-HUVECs
**TNF-α +<br>LA 0.1 mM**	71.4 ± 29.4 *	97.1 ± 21.8 ** #	* < 0.01 vs Basal C-HUVECs<br>** < 0.005 vs Basal GD-HUVECs<br># < 0.05 vs TNF-α GD-HUVECs
**TNF-α +<br>LA 0.2 mM**	76.2 ± 8.1 * ‡	60.4 ± 20.5 #	* < 0.01 vs Basal C-HUVECs<br>‡ < 0.05 vs TNF-α C-HUVECs<br># < 0.05 vs TNF-α GD-HUVECs
**TNF-α +<br>anti-VCAM-1**	65.9 ± 14.9 * ‡	57.7 ± 4.9 #	* < 0.01 vs Basal C-HUVECs<br>‡ < 0.05 vs TNF-α C-HUVECs<br># < 0.05 vs TNF-α GD-HUVECs
**TNF-α +<br>anti-VCAM-1**	80.2 ± 4.2 * ‡	71.26 ± 5.2 #	* < 0.01 vs Basal C-HUVECs<br>‡ < 0.05 vs TNF-α C-HUVECs<br># < 0.05 vs TNF-α GD-HUVECs

Table S3. Values and significance of Figure 3

Panel A	C-HUVECs	GD-HUVECs	p
**Basal**	0.95 ± 0.06	1.32 ± 0.19	n/a
**TNF-α**	11.5 ± 0.87 *	20.8 ± 1.11 ** ‡	* < 0.001 vs Basal C-HUVECs<br>** < 0.001 vs Basal GD-HUVECs<br>‡ < 0.05 vs TNF-α C-HUVECs
**TNF-α + Myo 0.1 mM**	10.48 ± 1.23 *	18.37 ± 2.07 **	* < 0.001 vs Basal C-HUVECs<br>** < 0.001 vs Basal GD-HUVECs
**TNF-α + Myo 0.5 mM**	9.84 ± 0.67 *	17.67 ± 1.87 **	* < 0.001 vs Basal C-HUVECs<br>** < 0.001 vs Basal GD-HUVECs
**TNF-α + Myo 1 mM**	9.49 ± 1.06 *	16.98 ± 1.03 ** #	* < 0.001 vs Basal C-HUVECs<br>** < 0.001 vs Basal GD-HUVECs<br># < 0.05 vs TNF-α GD-HUVECs
**TNF-α + LA 0.1 mM**	9.74 ± 0.9 *	16.28 ± 2.21 **	* < 0.001 vs Basal C-HUVECs<br>** < 0.001 vs Basal GD-HUVECs
**TNF-α + LA 0.2 mM**	7.35± 0.63 * ‡	13.36 ± 2.25 ** #	* < 0.001 vs Basal C-HUVECs<br>** < 0.001 vs Basal GD-HUVECs<br>‡ < 0.05 vs TNF-α C-HUVECs<br># < 0.05 vs TNF-α GD-HUVECs
**Panel B**	**C-HUVECs**	**GD-HUVECs**	**p**
**Basal**	2.49 ± 0.39	2.99 ± 0.34	n/a
**TNF-α**	9.12 ± 0.79 *	11.12 ± 0.7 ** ‡	* < 0.001 vs Basal C-HUVECs<br>** < 0.001 vs Basal GD-HUVECs<br>‡ < 0.05 vs TNF-α C-HUVECs
**TNF-α + Myo 0.1 mM**	8.26 ± 0.7 *	10.81 ± 0.48 **	* < 0.001 vs Basal C-HUVECs<br>** < 0.001 vs Basal GD-HUVECs
**TNF-α + Myo 0.5 mM**	8.52 ± 0.86 *	9.57 ± 0.67 **	* < 0.001 vs Basal C-HUVECs<br>** < 0.001 vs Basal GD-HUVECs
**TNF-α + Myo 1 mM**	7.53 ± 1.32 *	9.01 ± 0.64 ** #	* < 0.001 vs Basal C-HUVECs<br>** < 0.001 vs Basal GD-HUVECs<br># < 0.05 vs TNF-α GD-HUVECs
**TNF-α + LA 0.1 mM**	8.92 ± 1.07 *	8.22 ± 1.02 ** #	* < 0.001 vs Basal C-HUVECs<br>** < 0.001 vs Basal GD-HUVECs<br># < 0.05 vs TNF-α GD-HUVECs
**TNF-α + LA 0.2 mM**	6.99 ± 0.11 * ‡	7.86 ± 0.19 ** #	* < 0.001 vs Basal C-HUVECs<br>** < 0.001 vs Basal GD-HUVECs<br>‡ < 0.05 vs TNF-α C-HUVECs<br># < 0.05 vs TNF-α GD-HUVECs

Table S4. Values and significance of Figure 4

	C-HUVECs	GD-HUVECs	p
**Basal**	1.0 ± 0.0	1.28 ± 0.17	n/a
**H_2O_2**	1.67 ± 0.28	3.0 ± 0.44 * ‡	* < 0.005 vs Basal GD-HUVECs<br>‡ < 0.005 vs H_2O_2 C-HUVECs
**H_2O_2 + Myo 0.1 mM**	1.52 ± 0.25	2.42 ± 0.26 *	* < 0.005 vs Basal GD-HUVECs
**H_2O_2 + Myo 0.5 mM**	1.41 ± 0.15	2.02 ± 0.73	n/a
**H_2O_2 + Myo 1 mM**	1.36 ± 0.14	1.57 ± 0.48 #	# < 0.05 vs H_2O_2 GD-HUVECs
**H_2O_2 + LA 0.1 mM**	1.57 ± 0.11	1.89 ± 0.65 #	# < 0.05 vs H_2O_2 GD-HUVECs
**H_2O_2 + LA 0.2 mM**	1.27 ± 0.02	1.77 ± 0.31 #	# < 0.05 vs H_2O_2 GD-HUVECs

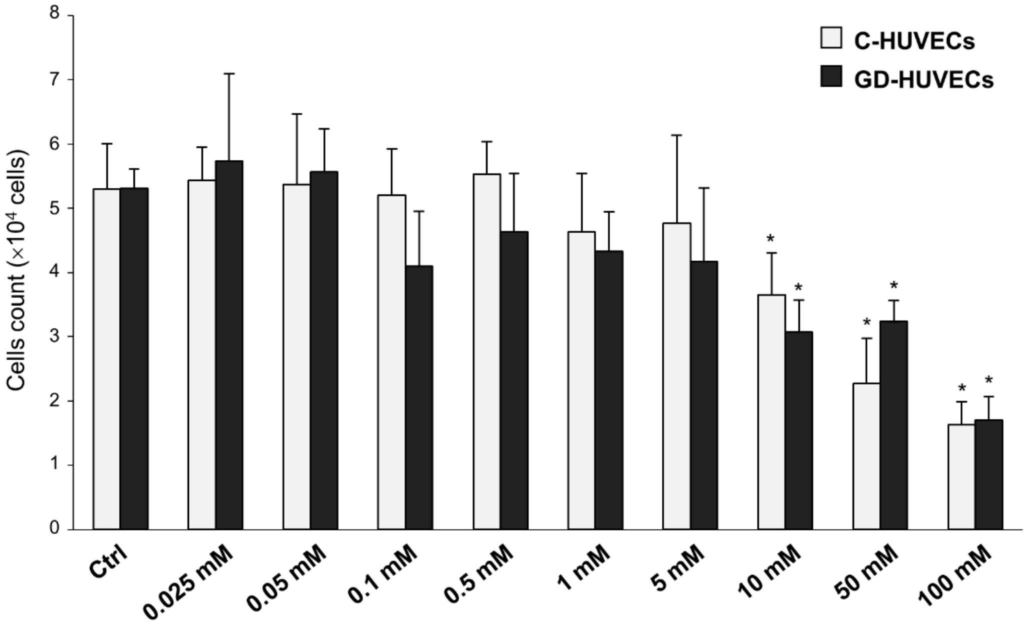

Figure S1. Effect of *in vitro* treatment with Myo on viability in C- and GD-HUVECs. $*p < 0.05$ in C- and GD-HUVECs vs Control (Ctrl) condition.
